# Embolization with Quick-Soluble Gelatin Sponge Particles for Lower Gastrointestinal Bleeding: A Multicenter Study

**DOI:** 10.3390/medicina61111964

**Published:** 2025-10-31

**Authors:** Chang Ho Jeon, Seung Boo Yang, Woo Jin Yang, Ji Hoon Shin, Kyu-Pyo Kim, Jung-Hoon Park, Jin-Hyoung Kim

**Affiliations:** 1 Department of Radiology, Eunpyeong St. Mary’s Hospital, College of Medicine, The Catholic University of Korea, Seoul 03312, Republic of Korea; changho.jeon@gmail.com; 2 Department of Radiology, Nowon Eulji University Hospital, College of Medicine, Eulji University, Seoul 01753, Republic of Korea; ysbysb28@gmail.com; 3 Department of Radiology, Korea University Guro Hospital, College of Medicine, Korea University, Seoul 08308, Republic of Korea; idleidiot@naver.com; 4 Department of Radiology and Research Institute of Radiology, Asan Medical Center, College of Medicine, University of Ulsan, Seoul 05505, Republic of Korea; jhparkz1125@gmail.com (J.-H.P.); m1fenew@daum.net (J.-H.K.); 5 Department of Oncology, Asan Medical Center, College of Medicine, University of Ulsan, Seoul 05505, Republic of Korea; kkp1122@gmail.com

**Keywords:** gastrointestinal bleeding, transarterial embolization, embolization, embolotherapy, bowel infarction

## Abstract

*Background and Objectives:* Transarterial embolization (TAE) serves as a valuable alternative for gastrointestinal bleeding when endoscopy fails or is inaccessible. Quick-soluble gelatin sponge particles (QS-GSPs) dissolve rapidly and may reduce ischemic complications compared to permanent embolic agents. This study evaluated the safety and effectiveness of TAE using QS-GSPs for acute lower gastrointestinal bleeding. *Materials and Methods:* This retrospective multicenter study analyzed patients who underwent TAE with QS-GSPs for acute nonvariceal lower GI bleeding between 2021 and 2024. Technical success (occlusion or stasis of blood flow in the target artery), clinical success (cessation of bleeding symptoms with hemodynamic stability during the week following TAE without major complications), and procedure-related complications were assessed. *Results:* A total of 29 patients (mean age 64.9 years) were included. Active bleeding was detected in 6 patients (20.7%) on angiography. Embolized arteries included jejunal (*n* = 7), ileal (*n* = 7), ileocolic anastomotic (*n* = 1), cecal (*n* = 2), colic (*n* = 7), and rectosigmoid (*n* = 5) arteries. QS-GSPs (150–350 μm (*n* = 10) or 350–560 μm (*n* = 19)), which dissolve completely within several hours, were used as the sole embolic agents. Technical and clinical success rates were 100% and 75.9% (22/29), respectively. Clinical failure occurred in seven patients (24.1%) due to persistent (*n* = 4) or recurrent (*n* = 3) bleeding within one week. Transient bowel ischemia occurred in two patients (6.9%) but resolved spontaneously. The clinical success rate did not differ significantly between patients with active bleeding (66.7%) versus those without (73.9%). *Conclusions:* TAE with QS-GSPs for acute lower GI bleeding demonstrated a favorable safety profile with clinical success exceeding 75%. Transient bowel ischemia occurred in 6.9% of patients with spontaneous resolution, and no bowel infarction was observed.

## 1. Introduction

Gastrointestinal (GI) bleeding is a common and clinically significant emergency that frequently requires hospitalization [1]. Upper GI bleeding (UGIB) and lower GI bleeding (LGIB) differ in typical etiologies, diagnostic yield, and natural history, although both carry substantial morbidity despite advances in resuscitation and endoscopy [1,2]. A recent worldwide systematic review reported that LGIB incidence ranges from ≈20.5 to 87 per 100,000 person-years, with mortality ≈0.8–3.5 per 100,000 person-years and case-fatality ≈0.5–8%, highlighting between-study heterogeneity but providing a global frame of reference [3]. Diverticular bleeding is the leading etiology of LGIB in adults, accounting for roughly one-third to two-thirds of cases across cohorts, though proportions vary by population and methodology [4].

Standard management prioritizes hemodynamic stabilization and correction of coagulopathy, followed by timely endoscopic evaluation and therapy when feasible [1,2]. For UGIB, guideline panels suggest endoscopy within 24 h and recommend transarterial embolization (TAE) when endoscopic hemostasis fails [1]. For LGIB, contemporary guidance emphasizes risk stratification, an increasing role for CT angiography in severe ongoing bleeding, and a non-urgent inpatient colonoscopy strategy in most stabilized cases [2,5]. Surgery is generally reserved for refractory scenarios—ongoing instability or recurrent hemorrhage despite optimal endoscopic and/or radiologic therapy—or when a specific pathology mandates operative control [1,5].

TAE is an image-guided endovascular procedure that achieves hemostasis by selectively occluding the culprit gastrointestinal arterial branch with embolic agents [6,7,8]. Indications include failure of or infeasibility for endoscopic hemostasis; computed tomographic angiography (CTA) or angiographic evidence of active bleeding or a culprit vessel; ongoing severe hemorrhage with hemodynamic instability; and the need for a nonsurgical option in high-risk surgical candidates [1,2,5,6,7,8]. In life-threatening hemorrhage, no absolute contraindications apply; relative contraindications include established transmural bowel ischemia requiring resection, inability to obtain vascular access, uncorrectable coagulopathy, severe iodinated-contrast allergy without viable alternatives, and advanced renal failure when contrast exposure cannot be mitigated [5,6,7,8]. The safety profile of TAE depends on embolic material and vascular territory: because collateral networks are relatively sparse in the lower GI tract, bowel ischemia risk is higher in LGIB than in UGIB and increases with more proximal or extensive occlusion—making embolic choice central to balancing durable hemostasis against ischemic complications [6,7,8].

Quick-soluble gelatin sponge particles (QS-GSPs) are resorbable particles engineered to provide temporary arterial occlusion, enabling hemostasis while potentially limiting prolonged ischemia [9]. Upon contact with blood or saline, QS-GSPs are reported to dissolve within hours, restoring perfusion after a short hemostatic window [9,10]. In the lower GI tract—where ischemic risk is a key constraint—a short-lived occlusion may stabilize bleeding while mitigating ischemic injury [6,7,8]. Against this background, we conducted a multicenter study evaluating the safety and effectiveness of TAE using QS-GSPs for acute nonvariceal LGIB, regardless of angiographic visualization of active extravasation. We hypothesized that QS-GSPs would achieve clinically acceptable bleeding control with a low incidence of ischemic complications in this ischemia-prone vascular territory.

## 2. Materials and Methods

### 2.1. Study Design and Ethics

This was a multicenter, retrospective study conducted at four tertiary hospitals. The protocol adhered to the Declaration of Helsinki. Institutional review board approval was obtained at all participating centers with a waiver of informed consent due to the retrospective design.

### 2.2. Patient Selection and Indications for TAE

#### 2.2.1. Inclusion

Consecutive adult patients who underwent TAE using quick-soluble gelatin sponge particles (QS-GSPs) as the sole embolic material for acute nonvariceal lower gastrointestinal bleeding (LGIB) between September 2021 and November 2024 were eligible. Acute nonvariceal GI bleeding was defined as hematemesis, melena, or hematochezia from a nonvariceal source occurring within 24 h prior to angiography.

#### 2.2.2. Exclusion

We excluded (1) variceal bleeding; (2) upper GI bleeding without a lower GI source; (3) cases managed without TAE; (4) TAE performed with embolic agents other than QS-GSPs or in combination as the initial procedure; and (5) insufficient clinical or imaging records to assess outcomes.

#### 2.2.3. Indications for TAE

TAE was performed if one or more of the following criteria were met despite initial resuscitation and standard care (including endoscopy when feasible): (1) active contrast extravasation or a definitive culprit lesion on CT angiography or endoscopy; (2) hemodynamic instability (systolic blood pressure < 90 mmHg) or ongoing transfusion requirement; (3) hemoglobin < 8.0 g/dL or ≥2 g/dL drop within 24 h with ongoing bleeding suspicion; (4) failure or infeasibility of endoscopic therapy; or (5) high surgical risk or anticipated delay to operative control.

### 2.3. Clinical Pathway and Diagnostic Work-Up

**Figure 1:**
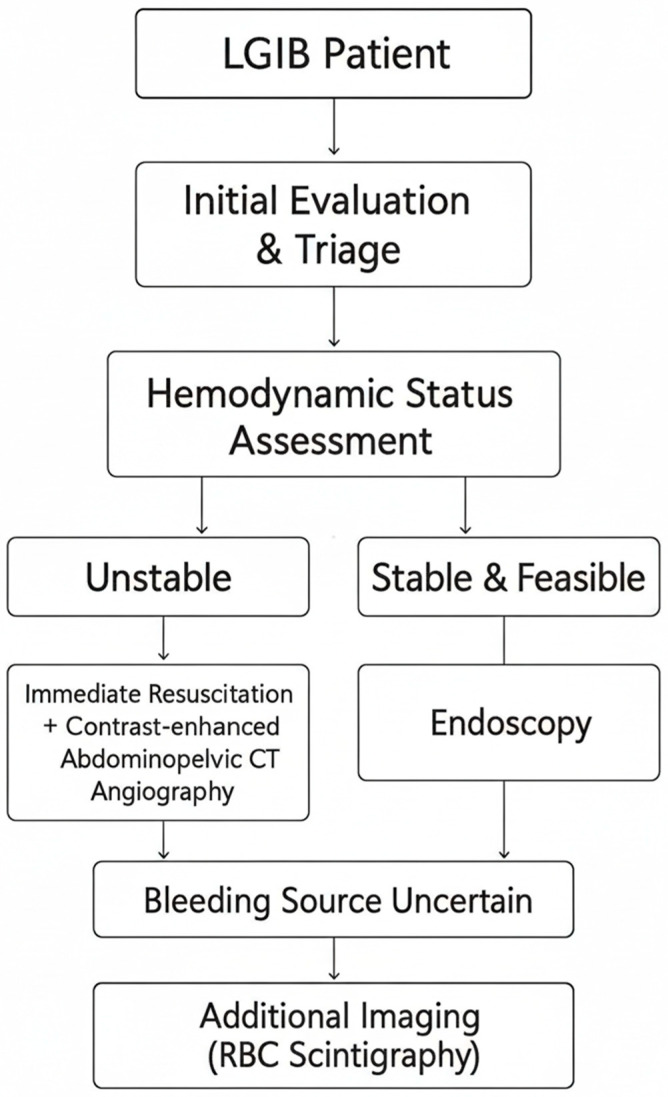
Clinical pathway for initial evaluation and triage of acute lower gastrointestinal bleeding.

Initial evaluation followed a standardized LGIB pathway across participating centers (Figure 1). Briefly, patients were triaged by hemodynamic status. When unstable, immediate resuscitation proceeded in parallel with contrast-enhanced abdominopelvic CT angiography; when stable and feasible, endoscopy was considered first. If the bleeding source remained uncertain, additional imaging such as RBC scintigraphy was performed at the discretion of the treating team.

In this acute LGIB cohort, capsule endoscopy was not employed. The diagnostic approach across centers prioritized rapid localization with computed tomography angiography (CTA) and/or colonoscopy, followed—when endoscopic hemostasis was not feasible or unsuccessful—by TAE. This strategy is consistent with contemporary guidelines, which recommend CTA for hemodynamically significant ongoing bleeding and reserve capsule endoscopy for clinically stable patients with suspected small-bowel bleeding after negative upper and lower endoscopy [2,11,12].

### 2.4. Angiography and Embolization Technique

All TAE procedures were performed in an interventional radiology suite by more than ten interventional radiology specialists from the four participating institutions. The operators had 5 to 27 years of experience, with a median of 12 years (interquartile range, 7.5–21 years). Vascular access was via a 5-F sheath in the common femoral artery. A 5-F catheter (e.g., RH catheter; Cook, Bloomington, IN, USA) over a 0.035-inch guidewire (Radifocus; Terumo, Tokyo, Japan) was used for mesenteric angiography, followed by superselective catheterization using 2.0–2.4-F microcatheters (e.g., Progreat; Terumo, or Radiomate; S&G Biotech, Yongin, Republic of Korea). QS-GSPs (K-IPZA^®^; Engain, Hwaseong, Republic of Korea) were the sole embolic agent; particles (150–350 μm or 350–560 μm) dissolve completely within 2~6 h upon contact with saline or blood.

When active extravasation was present but superselection was unfeasible, embolization was performed from the most proximal safe position; if no extravasation was observed, prophylactic embolization targeted the suspected site based on CT or endoscopy.

### 2.5. Definitions and Data Collection

#### 2.5.1. Definitions

Coagulopathy was defined as prothrombin time-international normalized ratio (PT-INR) > 1.5 or platelet count < 50,000/µL [13]. Hemodynamic instability was defined as systolic blood pressure < 90 mmHg [14]. Technical success was occlusion or stasis of target-artery flow on post-embolization angiography. Clinical success was cessation of bleeding symptoms with hemodynamic stability during the first week after TAE without major complications. Clinical failure was defined as the presence of one or more of the following three conditions within one week after the initial TAE: persistent bleeding (ongoing bleeding despite TAE), recurrent bleeding at the same site within one week after the initial successful hemostasis achieved by TAE, and procedure-related major complications. Complications were classified according to Society of Interventional Radiology guidelines.

#### 2.5.2. Data Collection

From electronic medical records and imaging archives, we obtained baseline characteristics, clinical presentation, bleeding etiology/site, prior hemostatic therapy, angiographic findings, procedural details, and outcomes. Additional variables included antithrombotic use, transfusion requirements, and operator experience.

### 2.6. Statistical Analysis

Continuous variables were summarized as the mean ± standard deviation (SD), median and range, and categorical variables were summarized as counts and percentages. Pre- and post-TAE hemoglobin levels were compared with the Wilcoxon signed-rank test, and clinical success rates between patients with and without angiographic extravasation were compared with Fisher’s exact test. A *p*-value < 0.05 was considered statistically significant. Analyses were performed using SPSS Statistics, version 23 (IBM Corp., Armonk, NY, USA).

## 3. Results

### 3.1. Patient Characteristics

The study included 29 patients (20 men; mean age, 64.9 ± 12.9 years; range, 21–82 years), and their baseline characteristics are summarized in Table 1. The primary clinical manifestations included hematochezia (*n* = 23), melena (*n* = 4), and both (*n* = 2). Bleeding sites were identified in the jejunum (*n* = 7), ileum (*n* = 7), ileocolic anastomosis (*n* = 1), cecum (*n* = 2), colon (*n* = 7), and rectosigmoid region (*n* = 5). The underlying causes of bleeding included: diverticular bleeding (*n* = 6), ulceration (*n* = 5), metastatic (*n* = 2) or lymphomatous (*n* = 2) involvement of the jejunum or ileum, postoperative bleeding (*n* = 1), colitis (*n* = 1), hemorrhoidal bleeding (*n* = 1), graft-versus-host disease (*n* = 1), and an indeterminate cause (*n* = 10).

The diagnosis of lower GI bleeding was established using endoscopy (*n* = 10), CT imaging (*n* = 13), or a combination of both (*n* = 5). In one patient, bleeding was detected via a red blood cell (RBC) scan. Six patients had a history of prior GI bleeding treatments, including endoscopic clipping with or without epinephrine injection (*n* = 4), TAE (*n* = 1), and small bowel resection with TAE (*n* = 1).

The median time between GI bleeding onset and TAE intervention was 1 day (range, 1–10 days). Coagulopathy was observed in three patients, and four patients experienced hemodynamic instability. Peri-procedural RBC transfusion amounted to a median of 5 units (IQR, 2–9) across the cohort (mean, 6.93 ± 5.71; range, 0–22).

### 3.2. Details of TAE

**Figure 2:**
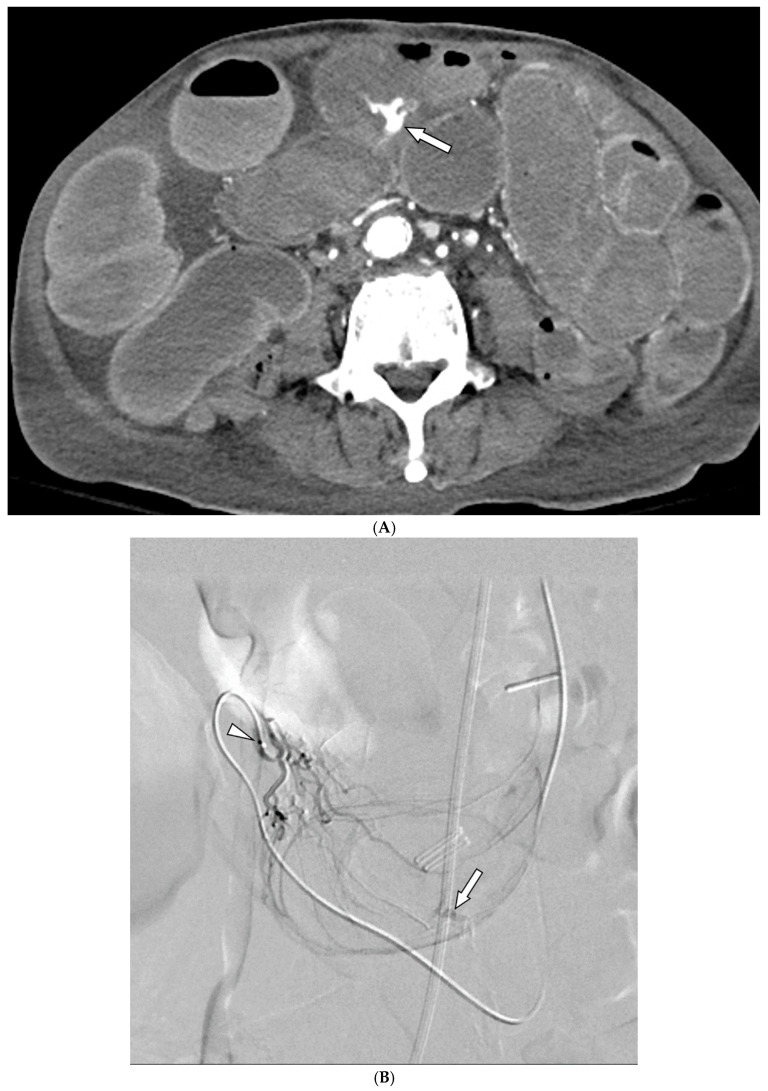

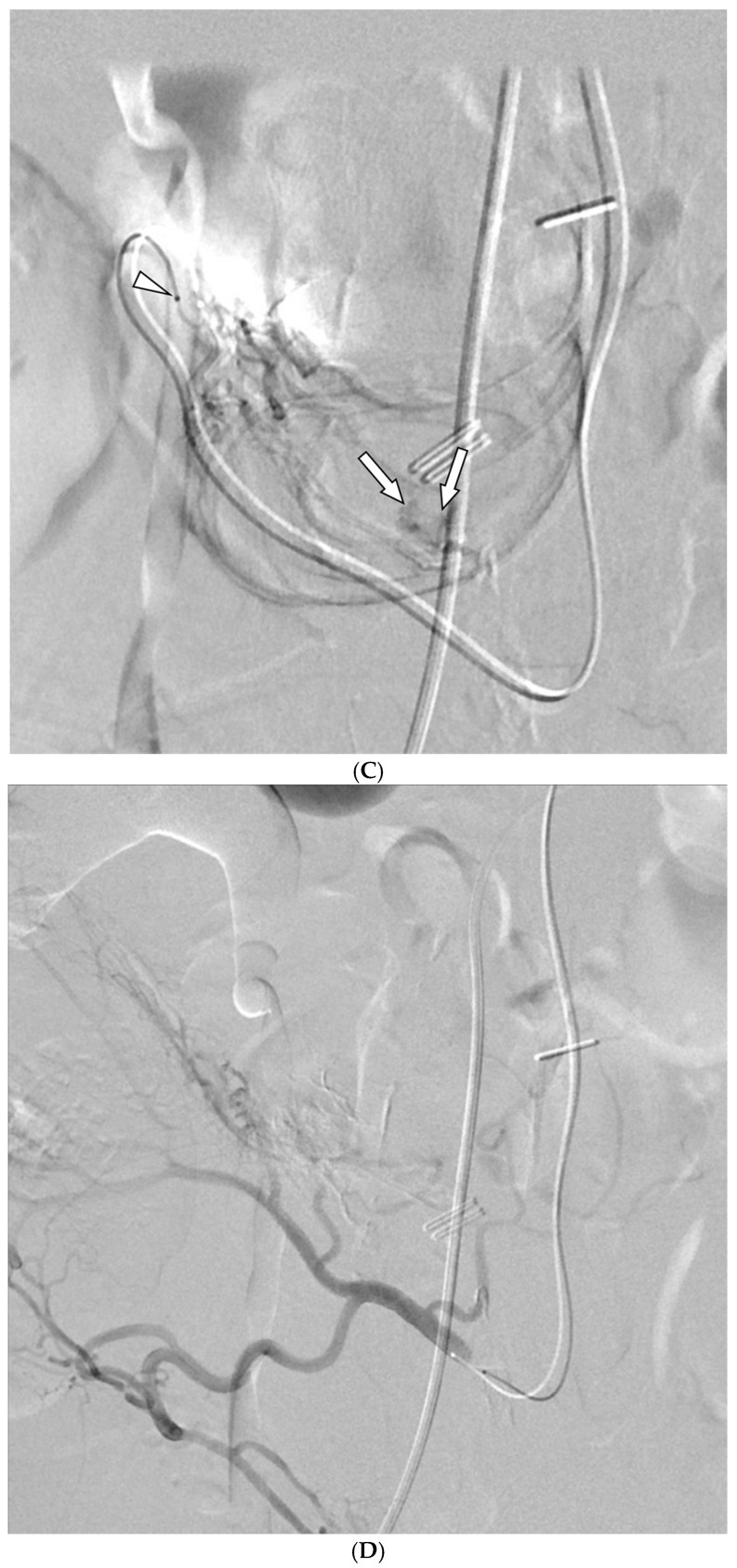
A 78-year-old man presented with hematochezia. (**A**) Contrast-enhanced axial CT image shows luminal contrast extravasation (arrow) at the ileal loop. (**B**,**C**) Selective ileal arteriography shows contrast extravasation (arrows). Due to the distance and tortuosity of the path from the microcatheter tip (arrowheads) to the bleeding focus, superselection was not possible; therefore, embolization was performed using quick-soluble gelatin sponge particles (350–560 µm). (**D**) Post-embolization arteriography demonstrates that the bleeding focus and the arterial branches leading to it are no longer visible. No further bleeding occurred during the 1-year follow-up.

**Figure 3:**
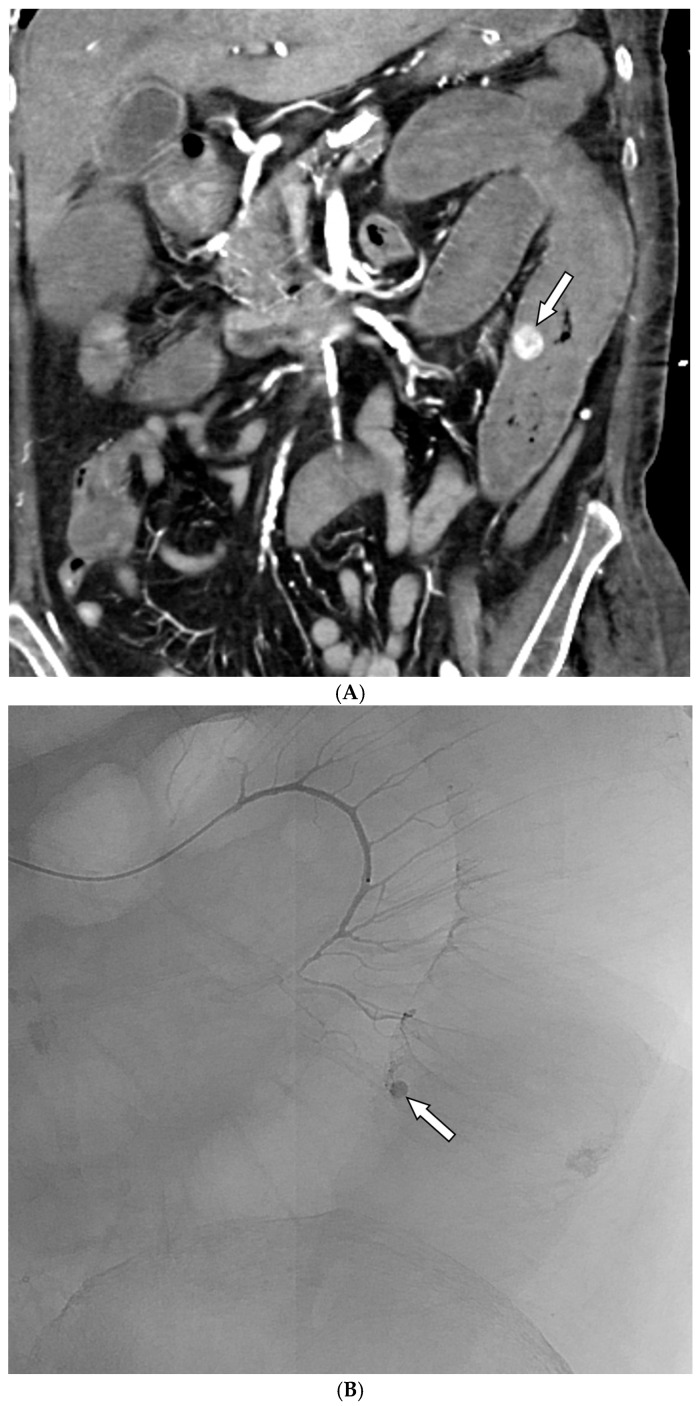

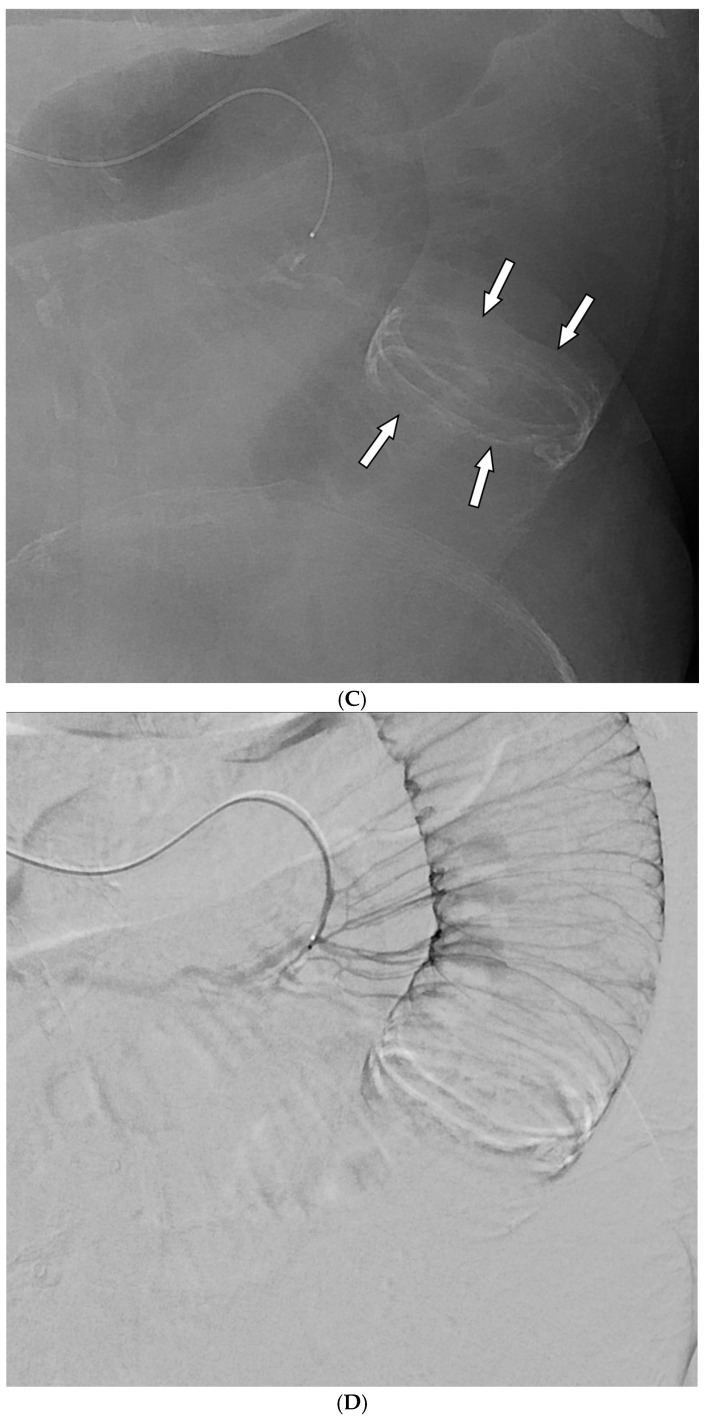
A 67-year-old woman presented with hematochezia. (**A**) Contrast-enhanced coronal CT image shows luminal contrast extravasation (arrow) at the jejunal loop. (**B**) Selective jejunal arteriography shows contrast extravasation (arrow). (**C**) Several levels of the vasa recta (arrows) were embolized with quick-soluble gelatin sponge particles (150–350 µm). (**D**) Post-embolization arteriography shows devascularization of the previously embolized vasa recta. No further bleeding occurred before the patient’s death one month after embolization.

**Figure 4:**
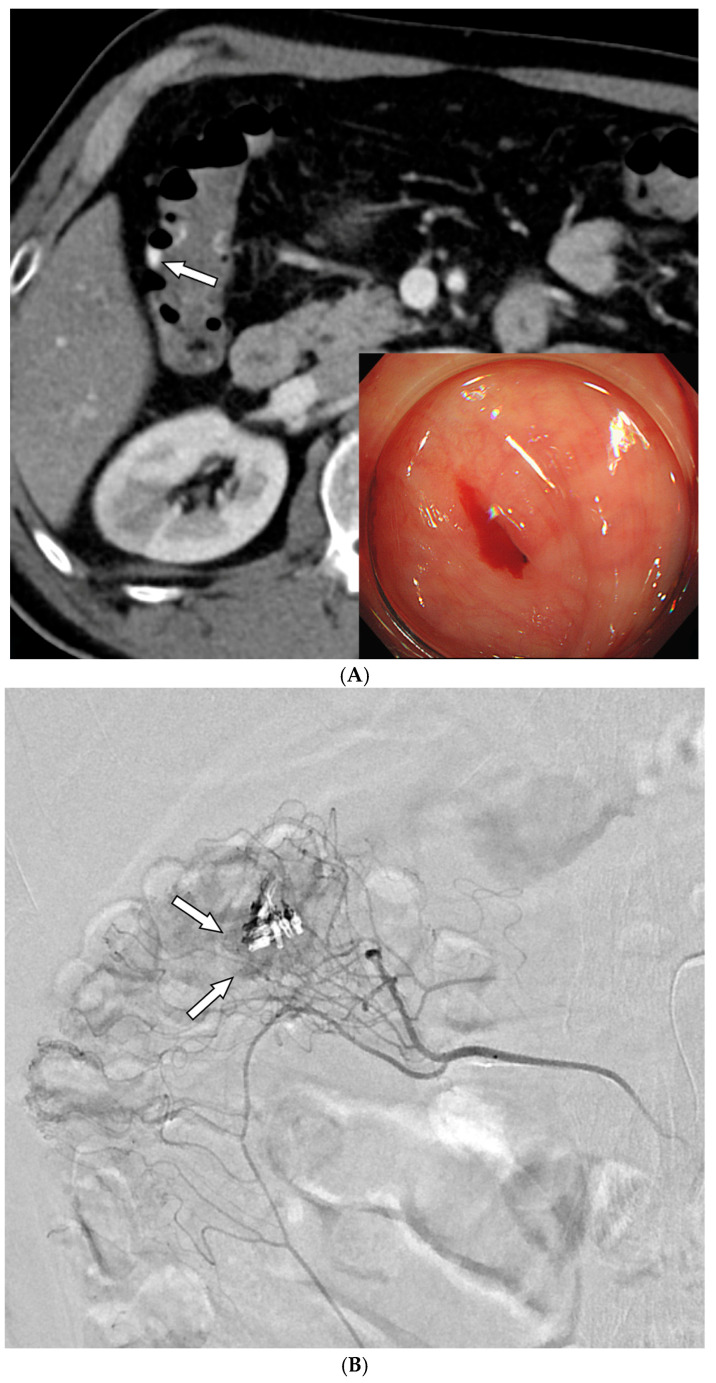

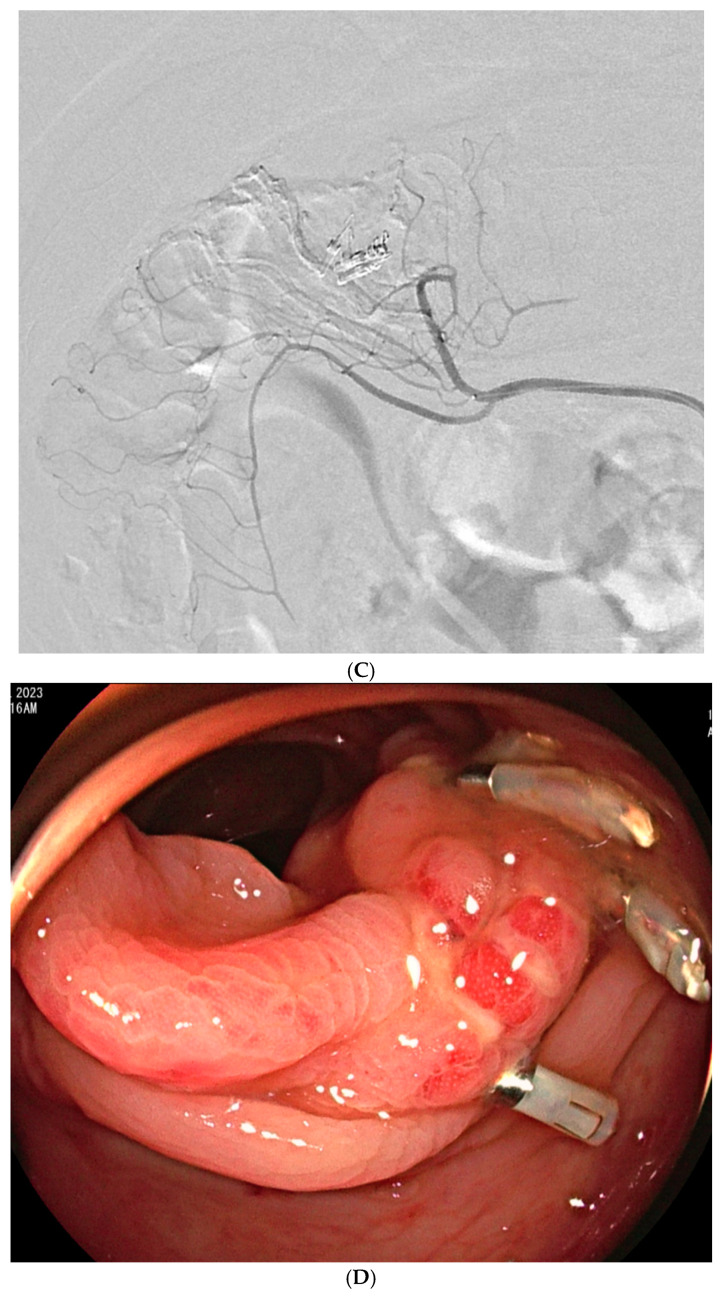
A 59-year-old man with colonic diverticula presented with hematochezia. (**A**) Contrast-enhanced axial CT image shows colonic diverticula with luminal contrast extravasation (arrow) at the hepatic flexure. Sigmoidoscopy performed one day after the CT scan reveals a diverticulum with bleeding (inset). Endoscopic hemoclipping was performed. (**B**) Superior mesenteric arteriography demonstrates focal hypervascularity (arrows) around the endoclipping site, without evidence of contrast extravasation. As superselection was not possible, the arterial branches leading to the endoclipping site were embolized using quick-soluble gelatin sponge particles (150–350 µm). (**C**) Post-embolization arteriography shows marked reduction in the previously observed hypervascularity around the endoclipping site. (**D**) Sigmoidoscopy performed two days after embolization reveals several ulcers and erythematous changes around the hemoclipping site, consistent with ischemic colitis. Associated abdominal pain resolved within three days. No further bleeding occurred during the 6-month follow-up.

Among the 29 patients, active bleeding was identified on angiography in six patients (20.7%, 6/29), and tumor staining was observed in two patients (6.9%, 2/29). However, the bleeding focus involved tortuous or small vessels that could not be selectively catheterized; therefore, QS-GSPs were administered from a more proximal location (Figure 2 and Figure 3). No evidence of bleeding or tumor staining was detected on angiography in the remaining 21 patients, where the target artery for embolization was determined based on endoscopic and/or CT findings, with angiography serving as a reference (Figure 4).

Embolization was performed on the following arteries: jejunal (*n* = 7), ileal (*n* = 7), ileocolic anastomotic (*n* = 1), cecal (*n* = 2), colic (*n* = 7), and rectosigmoid (*n* = 5). QS-GSPs (150–350 μm, *n* = 10; 350–560 μm, *n* = 19) were used as the sole embolic agents in all patients. Angiography following TAE confirmed occlusion or stasis of flow in the target artery in all patients, resulting in a 100% technical success rate.

### 3.3. Outcomes of TAE

During a follow-up period of 1 to 34 months (median, 4.5 months) post-TAE, clinical success was achieved in 22 patients (75.9%, 22/29), while clinical failure was observed in seven (24.1%, 7/29), including four with persistent bleeding after TAE and three with recurrent bleeding at the same site two, four-, and five-days post-TAE. Details of seven patients with clinical failure are provided in Table 2.

Procedure-related complications, including transient bowel ischemia, were observed in two patients and were classified as minor procedure-related complications. (6.9%, Figure 4). Transient ischemia of the rectum or ascending colon was confirmed via colonoscopy one and two days post-TAE, respectively. However, the transient ischemia of the rectum improved on endoscopy after three days, and the abdominal pain associated with ischemia of the ascending colon resolved within three days.

Among the six patients with active bleeding on angiography, clinical success was achieved in four (66.7%), while among the 23 patients without active bleeding, clinical success was observed in 17 (73.9%). No significant difference was found between the groups (*p* = 1). The median hemoglobin level increased from 7.4 ± 1.73 to 9.5 ± 1.22 g/dL (*p* < 0.05).

## 4. Discussion

A prior preliminary report assessed QS-GSP embolization for angiographically negative upper and lower GI bleeding. In that 10-patient series, technical success was 100% and clinical success (no rebleeding over 1–28 months) was achieved in 9/10 (90%). No bowel ischemic complications were observed [9]. However, the present study focuses on a larger cohort of patients with only lower GI bleeding, where the risk of bowel ischemic complications is expected to be higher. In this study, the rate of successful bleeding control without further bleeding during the week following TAE, referred to as clinical success, was 75.9%. These findings are consistent with those observed in a cohort of 112 patients with lower GI bleeding, where GSP was the primary embolic agent (administered to 20 of 24 patients), and the rebleeding rate was 25% [15]. A temporary occlusion by QS-GSPs seems sufficient to achieve effective hemostasis. While initial clot formation with platelet aggregation occurs rapidly within minutes, full stabilization, where the fibrin network reinforces the initial plug and secures the vessel against shear forces, typically develops over several hours. This suggests that a temporary occlusion lasting several hours, as provided by QS-GSPs, may be sufficient to allow the formation of a stable clot, particularly when compared to the longer durations required for irreversible mucosal ischemia.

QS-GSPs consist of medical-grade gelatin (denatured collagen) processed into porous, lightly cross-linked spheres [16]. Gelatin sponges have been in clinical use since the mid-20th century, and quick-soluble variants for TAE have been reported in interventional radiology since the 2010s [9,17]. Upon contact with blood or saline, the particles rapidly hydrate and provide mechanical hemostasis (platelet/fibrin scaffold) [17]. Subsequent hydration-driven matrix disintegration and proteolysis by circulating gelatinases restore flow [18]. In-vitro data indicate formulation-dependent dissolution (≈2–6 h at 37 °C), which may be accelerated by contrast mixing. During room-temperature preparation (~27 °C), partial mass loss over 2 h remains limited (2-h formulation < 40%, 6-h formulation < 30%) [16,18]. Particle size modulates kinetics. Smaller spheres (e.g., 150–350 µm) tend to hydrate/dissolve faster than larger ones (e.g., 350–560 µm) owing to higher surface-area-to-volume ratios [16,18].

Bowel ischemic complications, classified as minor procedure-related complications, were observed in only 2 of 29 patients (6.9%) in this study, and these complications resolved spontaneously within a few days. While reports on bowel ischemic changes following GSP-based TAE for lower GI bleeding are limited, one study reported that among 20 patients who received standard GSP embolization for lower GI bleeding, 1 patient (5%) died due to small bowel infarction and subsequent postoperative complications [15]. Another study reported that 4 of 27 patients (14.8%) with lower GI bleeding experienced bowel ischemic complications following standard GSP embolization [19]. In comparison, the 6.9% incidence of bowel ischemic changes observed in this study is acceptable. Furthermore, the spontaneous resolution within several days suggests that QS-GSPs are relatively safe, even in the lower GI tract, where collateral circulation is limited.

In contrast, animal experiments have demonstrated that occlusion of four or more vasa recta by NBCA increases the risk of bowel ischemic complications [20]. Despite the expectation that QS-GSPs, as a particulate embolic agent, may occlude a larger area, it resulted in fewer bowel ischemic changes, indicating that it is a highly safe agent for embolization in lower GI bleeding. Although ischemic complications in the small bowel may be difficult to confirm endoscopically, potentially leading to underreporting, major complications such as bowel infarction are clinically detectable, making it unlikely that severe complications would be underestimated.

The diameter of the arteries supplying the small bowel, as measured in cadaveric samples, ranges from 560 to 770 μm, while angiographic measurements range from 500 to 600 μm, yielding an approximate range of 500–800 μm [21,22]. The diameter of the arteries supplying the large bowel, as measured by angiography, ranges from 400 to 500 μm, which is slightly smaller than that of the small bowel [21]. Therefore, the particle size of QS-GSPs used in this study, which ranges from 150 to 560 μm, is considered appropriate for embolizing the vasa recta in both the small and large bowels. The absence of bowel infarction despite this particle size may be attributed to the resorbable nature of QS-GSPs, which dissolve within several hours. It is well established that complete acute occlusion of the enteric blood supply leads to irreversible mucosal ischemia within approximately six hours [23], suggesting that the dissolution of QS-GSPs within several hours makes permanent infarction unlikely. Consequently, even with embolization of relatively large areas involving multiple vasa recta, permanent infarction is not expected to occur. However, the optimal degradation time for temporary embolic agents remains unclear. Longer degradation times may reduce the risk of rebleeding due to recanalization but increase the risk of ischemic complications, whereas shorter degradation times have the opposite effect.

In a previous preliminary study, no patients with active bleeding were included [9]. However, in this study, 20.7% of patients presented with active bleeding. In cases of active bleeding, permanent embolic materials are typically used if superselective embolization is feasible. However, when arterial feeders are narrow or highly tortuous, making superselective embolization with a microcatheter unfeasible, traditional embolic agents may increase the risk of bowel ischemic complications. In contrast, QS-GSPs are expected to present a significantly lower risk of bowel ischemic complications, making it a promising option for active bleeding cases in which superselective embolization is not achievable. Furthermore, the lack of a significant difference in clinical success between the active bleeding and non-active bleeding groups in this study suggests that QS-GSPs may be a viable option even in cases of active bleeding.

This study had a few limitations. First, it was a retrospective study with a relatively small sample size. In particular, because our procedure-based dataset did not capture each center’s overall TAE volumes or the total number of GI-bleeding presentations, we could not determine the proportion of all eligible cases included. Consequently, potential selection and referral biases remain. Second, because the study was multicenter in design, the patient population was heterogeneous, and slight variations in the study protocol and procedural details may have existed between hospitals. Third, embolization-related adverse events, such as bowel ischemia or infarction, were primarily assessed through clinical records, and colonoscopy was performed only in selected patients after embolization to evaluate bowel ischemic complications.

## 5. Conclusions

In conclusion, TAE with QS-GSPs for acute lower GI bleeding was found to be safe, with a clinical success rate exceeding 75%. Transient bowel ischemia occurred in 6.9% of patients, but no cases of bowel infarction were observed. TAE with QS-GSPs may be particularly useful in cases where bleeding is suspected but the exact culprit vessel cannot be clearly identified, or in segments supplied predominantly by a single arterial branch.

## Figures and Tables

**Table 1:** Baseline characteristics of the study population (*n* = 29).

Characteristic	Category	*n* (Count)	%
Gender	Male (M)	20	69.0
	Female (F)	9	31.0
Age (years)	Mean 64.9 ± 12.9; Median 68; Range 21–82 years		
Underlying Disease *	Cardiovascular Diseases ^†^	12	41.4
	Malignancies/Cancers ^‡^	2	6.9
	Kidney/Metabolic Diseases ^§^	9	31.0
	Respiratory Diseases ^‖^	1	3.4
	Other Diseases ^¶^	2	6.9
	None/Unknown	6	20.7
Anticoagulant/Antiplatelet History	None	24	82.8
	Clopidogrel	2	6.9
	Direct Oral Anticoagulant (DOAC)	1	3.4
	Aspirin	1	3.4
	Aspirin + Clopidogrel	1	3.4

Notes: * Patients may have multiple underlying diseases; therefore, the total percentage exceeds 100%. ^†^ Cardiovascular Diseases: Major heart and vascular diseases (e.g., Hypertension, Heart Failure, Atrial Fibrillation, etc.). ^‡^ Malignancies/Cancers: Various solid and hematological cancers (e.g., Liver Cancer, Breast Cancer, Multiple Myeloma, etc.). ^§^ Kidney/Metabolic Diseases: Chronic Kidney Disease, Diabetes Mellitus. ^‖^ Respiratory Diseases: Chronic Obstructive Pulmonary Disease. ^¶^ Other Diseases: Behcet’s Disease, Pontine Infarct, etc.

**Table 2:** Characteristics of Seven Patients with Clinical Failure.

No./Sex/Age	Bleeding Cause	Embolized Arteries	Angiography	Embolic Materials	Rebleeding Onset	Management	Remark
1/F/81	Lymphoma	Jejunal a.	Active bleeding	QS-GSPs (350–560 μm)	Persistent	Jejunal resection the next day	Improved
2/F/72	Uncertain	Ileal a.	Active bleeding	QS-GSPs (350–560 μm)	Persistent	TAE with NBCA	Improved
3/F/49	Diverticular bleeding	Cecal a.	No bleeding	QS-GSPs (350–560 μm)	4 days later	Endoscopic clipping	Improved
4/M/59	Diverticular bleeding	Right colic a.	No bleeding	QS-GSPs (350–560 μm)	2 days later	TAE with QS-GSPs	Improved
5/M/70	Colitis	Superior rectal a.	No bleeding	QS-GSPs (350–560 μm)	5 days later	TAE with QS-GSPs and NBCA	Improved
6/M/71	Diverticular bleeding	Right colic a.	No bleeding	QS-GSPs (350–560 μm)	Persistent	Endoscopic clipping	Improved
7/F/70	Uncertain	Superior rectal a.	No bleeding	QS-GSPs (350–560 μm)	Persistent	No	Improved, transient ischemic colitis 1 day later

## Data Availability

The raw data supporting the conclusions of this article will be made available by the authors upon request.
